# To Dr. Bradley

**Published:** 1802-08-01

**Authors:** R. Langslow

**Affiliations:** Halesworth, Suffolk


### To Dr. BRADLEY.

SIR,

I Had previouſly to the appearance of your laſt Number, written down my ſentiments relative to the remuneration of Dr. Jenner, for his valuable diſcovery; in which, however, I am happy to find myſelf anticipated by ſo able a judge as the ingenious Dr. Beddoes. Sir, I ſhould wiſh the matter to be put in *this* point of view: Had Dr. Jenner, after making the Diſcovery, propoſed to publiſh a plan, which, if univerſally adopted, would in the courſe of ten or fifteen years exterminate from our iſlands ſo cruel and fatal a ſcourge as that of Small-pox, (leaving the proof of its efficacy to experiment, and the teſt of time) I have little doubt but the propoſition would have been embraced with avidity, and the ſum of fifty thouſand pounds have been conſidered as a ſmall remuneration to the author of ſo invaluable a bleſſing. What is the amount of ſuch a ſum divided amongſt the individuals of the populous iſlands of Great Britain and Ireland, at this time perhaps amounting to fourteen fourteen millions? A very little calculation will inform us, that this ſum is no greater than amounts to the demand of *nearly* one penny each; and let it be obſerved and remembered, that *every one* of theſe individuals, *without exception*, is nearly and dearly intereſted in this diſcovery; I cannot therefore but ſuppoſe, that the motion for the vote of ten thouſand pounds, was carried without *properly* adverting to the value of the Diſcovery, or the merit of the Diſcoverer, whoſe candour, ingenuouſneſs, and humanity, muſt ever reflect the greateſt honour on our nation.

Permit me, Sir, to add, that theſe Obſervations flow from the pen of one who had, for more than twenty-ſeven years, indulged a *favourite* theory on a *peculiar* mode of treating the inoculated Small-pox,* but who, from motives of conviction alone, and from the moment he *experimentally* diſcovered the *grand* ſuperiority of the Vaccine Inoculation, not only relinquiſhed his *own* opinions, but, which is ſtill more, ventured to recommend, at ſo early a period as in the year 1800, a National Parliamentary Reward.†

If theſe Hints ſhould tend to promote a reconſideration of the Reward granted Dr. Jenner, I ſhall feel myſelf highly gratified in the reflection of having placed the value of this Diſcovery in a *new* point of view.

I am, &c.

R. LANGSLOW, M. D.

*Haleſworth, Suffolk, July* 10, 1802.

---

### *Observations on Vaccine Inoculation; communicated by Mr.* THOMAS, *of Pimlico, Member of the Royal College of Surgeons.*

THE highly intereſting diſcovery and candid publication of Dr. Jenner, having undergone the ſeverity of criticiſm, ſtood the teſt of experience, and moreover, been ſtamped with the approbation of Parliament; it may be thought unneceſſary for any private individual to ſay more, with a view of rendering

 the

---

* Vide an Inaugural Diſſertation, publiſhed by the Author in 1791, at Glaſgow, entitled, "Obſervationes Experimentaque de Vi Tabis Variolocæ, in ſtadiis morbi diverſis; et de uſu Mercurii ad morbum mitigandum."—Sold by Callow, Crown Court, Soho.

† Vide page 132 of a Pamphlet, entitled, "The Caſe of Maſter Day, of Yoxford, &c." Sold by Murray and Highley, Fleet-ſtreet; and Callow, Crown-court, Soho.

